# Tubelight Adrenals in Diabetic Ketoacidosis

**DOI:** 10.5811/cpcem.2020.6.47617

**Published:** 2020-07-20

**Authors:** Prakrati Yadav, Akhilesh Kumar, Rohit Mathur, Pawan Garg, Maya Gopalakrishnan, Mahendra Kumar Garg

**Affiliations:** All India Institute of Medical Sciences, Department of internal Medicine, Jodhpur, India

**Keywords:** Diabetic ketoacidosis, acute pancreatitis, hypertriglyceridemia, CT hypoperfusion, tubelight adrenal

## Abstract

**Case Presentation:**

We report a patient with the triad of diabetic ketoacidosis, hypertriglyceridemia, and acute pancreatitis associated with computed tomography hypoperfusion complex and adrenal hyperdensity on abdominal imaging – an association not previously reported in diabetic ketoacidosis.

**Discussion:**

Presence of computed tomography hypoperfusion complex with hyperdense ‘Tubelight adrenals’ in a patient with diabetic ketoacidosis is associated with poor prognosis and thus serves to guide clinicians towards early and aggressive management.

## CASE PRESENTATION

A 27-year-old male with type 1 diabetes who was poorly compliant with insulin therapy presented to our emergency department (ED) with severe abdominal pain. His records revealed repeated ED visits for abdominal pain over the prior month. Based on laboratory findings the patient was diagnosed with diabetic ketoacidosis (DKA) (Table). Further evaluation demonstrated hypertriglyceridemia, elevated serum amylase, and elevated lipase.

Computed tomography (CT) with intravenous contrast showed findings consistent with acute pancreatitis as well as enhancing bilateral adrenal glands with mucosal hyperenhancement of bowel loops and narrow caliber of abdominal aorta with imperceptible inferior vena cava, suggesting hypoperfusion complex (Image). Despite aggressive management, the patient developed hypovolemic shock, metabolic acidosis worsened, and sensorium deteriorated. An abdominal drain was placed and he was intubated, mechanically ventilated, and subsequently managed in the intensive care setting. The patient expired the next day.

## DISCUSSION

The triad of diabetic ketoacidosis, hyperlipidaemia, and acute pancreatitis is important as it leads to profound hypovolemia comparable to post-traumatic shock, which leads to characteristic hypoperfusion complex on CT.^1^ In 1987 Taylor et al first described CT hypoperfusion complex in three children with post-traumatic shock with dilated bowel, enhancing bowel walls, pancreas, kidneys, aorta and inferior vena cava.^2^ Hyperdensity of normal-sized adrenal gland was later added to this complex by Sivit et al in paediatric patients who had sustained blunt abdominal trauma.^3^

**CPC-EM Capsule**

What do we already know about this clinical entity?

Diabetic ketoacidosis when associated with acute pancreatitis and hypertriglyceridemia results in profound hypovolemic shock. Computed tomography (CT) finding in such patients corresponds to post-traumatic shock known as ‘CT hypoperfusion complex.’

What is the major impact of the image(s)?

Profound hypovolemia may result in CT hypoperfusion complex and hyperdense adrenal, or “tubelight” (fluorescent tube-shaped) adrenals.” This CT finding indicates poor prognosis.

How might this improve emergency medicine practice?

Presence of this finding may guide physicians toward early and aggressive fluid management in these patients.

The finding of adrenal hyperdensity, which we describe as “tubelight adrenal sign” [fluorescent-tube shaped] in our patient as a part of CT hypoperfusion complex is unique as it has not been reported in the setting of DKA and is associated with increased mortality. Early imaging for diagnosis of pancreatitis and associated CT hypoperfusion complex with hyperdense tubelight adrenals can aid in guiding treatment and prognosis in these patients. Presence of tubelight adrenal sign on CT must alert the clinicians to possible adverse outcome and these patients should be initiated with early and aggressive fluid therapy.

## Figures and Tables

**Image:**
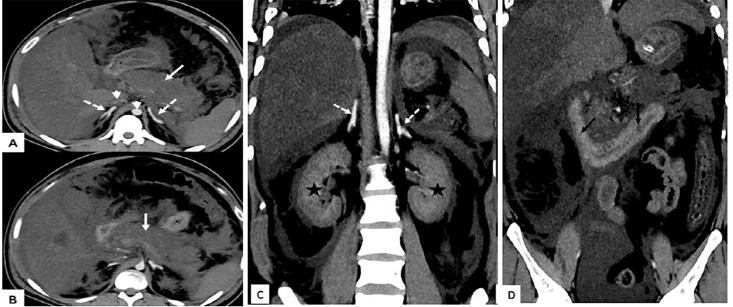
Contrast-enhanced computed tomography abdomen axial (A, B) and coronal (C, D) images showing diffuse pancreatic necrosis (white arrow) with significant peripancreatic inflammation. Intense enhancing bilateral adrenal glands (dashed arrow) with mucosal hyperenhancement of small bowel loops (black arrow) and gross ascites are visible. The short white arrow indicates narrow caliber of abdominal aorta with imperceptible inferior vena cava. Bilateral kidneys (asterisks) are heterogeneously enhancing with perinephric fat stranding likely due to diabetic nephropathy.

**Table:** Laboratory values of patient with poorly controlled diabetes type 1 at presentation.

Lab parameter	Value	Reference range
Blood glucose	442 mg/dl (25.08 mmol/L)	Below 200 mg/dl (Below 11.1 mmol/L)
Glycated hemoglobin (HbA1C)	11.4%	4.0–6.2%
Total leukocyte count with differentials	17.37 x 10 3/μL (N:79%, L:17 % M:3.2%)	< 11.0 x 103/ μL
Serum amylase	440 U/L	28–100 U/L
Serum lipase	1520 U/L	< 67 U/L
Serum cholesterol	770 mg/dl	Desirable: <200 mg/dL
Serum triglyceride	8210 mg/dl.	Normal: <150 mg/dL
Blood urea	20 mg/dL (7.14 mmol/L)	17–43 mg/dL
Serum creatinine	1.0 mg/dL (88.4μmol/L)	Male : 0.67–1.17 mg/dL Female : 0.51–0.95 mg/dL
pH	6.67	7.350–7.450
Serum bicarbonate	7.8 mmol/L	22–29 mmol/L
Anion gap	14	12 + 4
Urinary ketones	4+	Negative
Serum calcium	6.3 mg/dL (1.58 mmol/L)	8.8–10.6 mg/dL

*mg*, milligram; *dL*, deciliter; *mmol*, millimole; *L*, liter; *μL*, microliter; *N*, neutrophils; *L*, lymphocytes; *M*, monocytes; *U*, units; *μmol*, micromole.
